# What Can Epidemiological Studies Tell Us about the Impact of Chemical Mixtures on Human Health?

**DOI:** 10.1289/ehp.1510569

**Published:** 2016-01-01

**Authors:** Joseph M. Braun, Chris Gennings, Russ Hauser, Thomas F. Webster

**Affiliations:** 1 Department of Epidemiology, Brown University, Providence, Rhode Island, USA; 2 Department of Preventive Medicine, Icahn School of Medicine at Mount Sinai, New York, New York, USA; 3 Departments of Environmental Health and Epidemiology, Harvard T.H. Chan School of Public Health, Boston, Massachusetts, USA; 4 Department of Environmental Health, Boston University School of Public Health, Boston, Massachusetts, USA

## Abstract

Humans are exposed to a large number of environmental chemicals: Some of these may be toxic, and many others have unknown or poorly characterized health effects. There is intense interest in determining the impact of exposure to environmental chemical mixtures on human health. As the study of mixtures continues to evolve in the field of environmental epidemiology, it is imperative that we understand the methodologic challenges of this research and the types of questions we can address using epidemiological data. In this article, we summarize some of the unique challenges in exposure assessment, statistical methods, and methodology that epidemiologists face in addressing chemical mixtures. We propose three broad questions that epidemiological studies can address: *a*) What are the potential health impacts of individual chemical agents? *b*) What is the interaction among agents? And *c*) what are the health effects of cumulative exposure to multiple agents? As the field of mixtures research grows, we can use these three questions as a basis for defining our research questions and for developing methods that will help us better understand the effect of chemical exposures on human disease and well-being.

## Introduction

Biomonitoring studies confirm that humans are exposed to a large number of environmental chemicals across the life span, often simultaneously (CDC 2015; Woodruff et al. 2011). Although there is growing concern that exposure to chemical mixtures during critical periods of human development could increase the risk of adverse health effects including allergic diseases, cancer, neurodevelopmental disorders, reproductive disorders, and respiratory diseases, researchers primarily study chemicals as if exposure occurs individually. This one-chemical-at-a-time approach has left us with insufficient knowledge about the human health effects of exposure to chemical mixtures. Quantifying the risk of disease from environmental chemical mixtures could help identify modifiable exposures that may be amenable to public health interventions.

As interest in chemical mixtures evolves, there is a need for greater involvement of epidemiologists in this area of research (Carlin et al. 2013). We describe some of the unique challenges to studying environmental chemical mixtures in human populations and propose three broad questions related to chemical mixtures that epidemiology can address. We believe this information will help investigators select the best epidemiological and statistical methods for studying chemical mixtures in human populations and consider the limitations of these methods in their studies.

### Challenges to Studying Chemical Mixtures

*Measuring environmental chemical exposure.* Measuring human exposure to a large number of chemicals is a daunting task. First, the study of chemical mixtures requires accurate measurement of the individual components of the mixture. Sensitive and specific exposure biomarkers are one method to assess chemical exposures. These biomarkers have revolutionized the study of chemical mixtures by allowing investigators to directly measure individual chemical concentrations in a variety of biospecimens (Needham et al. 2008). While chemical exposure biomarkers have many strengths, caution should be exercised because of the potential limitations related to misclassification of exposures with high within-person variability (e.g., many short half-life chemicals such as bisphenol A), reverse causality due to pharmacokinetic factors (e.g., excretion) related to the outcome under study (Savitz 2014), or the inability for the biomarker to represent exposure during the etiologically relevant time period.

Second, epidemiologists who study mixtures must consider pragmatic factors when measuring a large number of environmental chemicals. Financial cost is perhaps the most important limiting factor when using biomarker-based approaches to study chemical mixtures because the inclusion of more components in targeted analytical chemistry methods increases the cost, often at the sake of sample size. In addition to cost, the volume of biospecimens (e.g., blood, urine, and plasma) required for these assays and the collection of samples from special populations (e.g., neonates or toddlers) must be considered. The streetlight effect, a type of observational bias, has limited the number of chemicals studied because epidemiologists have typically measured only a few chemicals, choosing from those known to be of concern or those for which measurement methods currently exist. However, advances in analytic chemistry methods (e.g., nontargeted analysis) allow epidemiologists to broaden their scope and identify new or replacement chemicals introduced into commerce and industry.

*Some statistical challenges.* The risk of false-positive results is a concern when analyzing a large number of exposures. Several statistical methods, including the Bonferroni correction, are used to reduce type I error rates in studies with a large number of hypotheses (Glickman et al. 2014). The Bonferroni approach is an appealing method when dealing with hundreds or thousands of potential hypotheses in studies of mixtures; however, over-reliance on significance testing in observational studies where exposures are not randomized and are often correlated with one another can be problematic (Poole 2001; Rothman 1986, 1990; Savitz 1993). Although hypothesis testing is still used as a method of inference, epidemiologists must also assess the validity, magnitude, and precision of observed associations rather than just the statistical significance of associations.

Type II errors can be equally problematic in studies of chemical mixtures. The statistical power to precisely estimate subtle effects between chemicals and human health may be limited by sample size, the accuracy of exposure assessment methods (e.g., nondifferential exposure misclassification), or multicollinearity issues due to correlations among chemicals in the mixture (i.e., inflated variance estimates) (Cox et al. 2015; Braun et al. 2014).

*Confounding due to correlated exposures.* While confounding due to socioeconomic factors associated with both the exposure and outcome is almost always considered as a potential source of bias in environmental epidemiology studies, confounding due to correlated copollutants can also exist. For example, in studies of persistent pollutants like polychlorinated biphenyls (PCBs), dioxins, and organochlorine pesticides, exposure biomarkers are often correlated with each other and may also be correlated with health outcomes (Longnecker et al. 2000). Such confounding, depending on the magnitude of correlation between the pollutants, can make identifying the effect of an individual chemical difficult, if not impossible. Thus, it is essential to understand the patterns of environmental exposures in human populations, as well as the correlation between individual agents, to determine if copollutant confounding may be present and whether public health interventions designed to reduce chemical exposures should target the entire mixture or components of it.

*Identifying important mixtures.* The pattern of human exposure to environmental chemicals is complex and multifactorial. Many pollutants are correlated with each other and some combinations of exposures are more likely than others. Because there is a need to identify patterns of exposure that are most likely to be relevant to human health, some pollutant combinations may be of less relevance if there are no individuals with a given pattern of exposure. Thus, in ranking the importance of these patterns, epidemiologists will need to consider the variability and prevalence of the exposure in the source population, the potential potency of the individual chemical components, and the ability to effectively reduce or mitigate the impact of exposure if adverse health effects are identified.

*Lack of standard methods to evaluate environmental mixtures.* A variety of statistical methods are available to address questions related to chemical mixtures (Billionnet et al. 2012; Sun et al. 2013), but there is no consensus on standard methods for studying environmental mixtures in epidemiological studies. Although we do not advocate for a formulaic approach, we believe it would be helpful to have a better understanding of the types of mixtures-related questions that epidemiologists can address so that appropriate methods and statistical tools can be selected to adequately address research and public health needs.

### Types of Questions Epidemiology Can Address

In this section, we describe three broad questions related to chemical mixtures that epidemiological studies could address; in Table 1, we list examples of how these questions have been addressed using different approaches, as well as the challenges to implementing them.

**Table 1:** Description and examples of questions related to chemical mixtures and human health that epidemiological studies can address.

Question	Examples and Methods	Challenges
What are the health effects of individual chemicals within a mixture?	Quantified the association between prenatal exposure to 52 endocrine-disrupting chemicals and children’s autistic behaviors using semi-Bayesian shrinkage methods (Braun et al. 2014). Used elastic net to examine the association between 16 prenatal exposures and birth weight (Lenters et al. 2015). Examined the association between 188 environmental factors and serum lipid levels using an environment-wide association study (Patel et al. 2012).	Some approaches may not adequately address copollutant confounding. Multiple comparisons. Disentangling the effect of highly correlated copollutants.
What are the interactions between chemicals within a mixture?	Determined if the neurotoxic effects of lead were greater among children with higher manganese exposure using product interaction terms (Claus Henn et al. 2012). Identified and examined interactions between multiple metal biomarkers and child mental development using Bayesian kernel machine regression (Bobb et al. 2015).	Difference in toxicologic and epidemiologic definitions of interaction (Howard and Webster 2013). Multiple comparisons. Imprecise effect estimates and reduced statistical power for detecting interactions.
What is the health effect of cumulative chemical exposure?	Examined the relationship between child anthropometry and exposure to dioxins using a toxic equivalency summary measure (Burns et al. 2011). Estimated the association between different chemical classes and non-Hodgkin lymphoma using weighted quantile sum regression (Czarnota et al. 2015). Used principal components analysis to examine the association between phthalate exposures and child anthropometry (Maresca et al. 2015).	Verifying the assumption of no interaction between individual components. Estimating cumulative exposure metrics for specific health outcomes. Availability of information to create biologically weighted summary measures. Interpretation of results from more complex statistical methods.

*What are the health effects of individual chemicals within a mixture?* The first question epidemiology can address is the association between individual chemical exposures in a mixture and human health outcomes. Because of the large number of environmental agents that humans are exposed to, there is a need to identify exposures that are most strongly associated with adverse health outcomes including individual exposures or groups of highly correlated and related exposures with a common source (e.g., Aroclors of PCB). The results of these studies would help guide public health efforts by allowing us to intervene on those agents that are most likely to be associated with human health.

There are several methods to quantify the association between individual chemical exposures and human health outcomes. An approach taken by many researchers is to quantify the association between each chemical exposure and the health outcome of interest in separate statistical models and then decide which are the most important (Patel et al. 2012). This approach can be extended by accounting for the correlated nature of copollutants and adjusting for potential confounding bias using hierarchical or Bayesian methods (Braun et al. 2014), as well as variable selection techniques such as weighted quantile sum (WQS) regression, elastic net, or least absolute shrinkage and selection operator (LASSO) (Czarnota et al. 2015; Lenters et al. 2015). Because of the correlated nature of many environmental pollutants, it is important to adjust for copollutant confounding using appropriate methods when trying to identify single exposures within a mixture that are most important to human health. Failure to do so could result in attributing one exposure to an adverse health outcome, when it might be due to another correlated copollutant.

*What are the interactions between chemicals within a mixture?* The second question epidemiological studies can address is whether two or more environmental chemical exposures have a greater than additive (i.e., synergistic) or subadditive (i.e., antagonistic) association with the health outcome of interest. For example, if we examine the risk of disease in relation to two binary exposures, then the standard epidemiological approach to interaction determines if the risk of disease among those exposed to both agents simultaneously is greater than the additive risk among those exposed to each agent individually. Two points are important to consider with interactions: First, even in the absence of a greater than additive interaction between two or more chemicals, joint exposure to these chemicals could have a cumulative effect (Howdeshell et al. 2015). Second, it is critical to note that toxicologists and epidemiologists define interaction differently. For instance, simple concentration-additive effects that are observed in toxicology experiments would be considered synergistic or antagonistic using epidemiological definitions when dose–response curves are nonlinear (Howard and Webster 2013).

Statistically examining interactions between chemicals would help identify synergies or antagonisms between exposures or determine if one or more exposure modifies the effect of other exposures. This could be approached agnostically using variable selection procedures (e.g., LASSO or elastic net) or Bayesian kernel machine regression (Bobb et al. 2015; Sun et al. 2013). Alternatively, a candidate approach could examine interactions between chemicals that act on common biological pathways related to the health outcome of interest. Two primary determinants of our ability to identify interactions will be sample size and the pattern of correlation between exposures. With a fixed sample size, it may be difficult to identify interactions between chemicals because the number of observations will diminish as smaller and smaller strata are examined for each additional chemical-by-chemical interaction considered. In addition, when two or more exposures are highly correlated, there may be an insufficient number of participants with exposure to only one of the agents, thus limiting our ability to examine the impact of only one exposure. Indeed, when exposures are highly correlated, their individual or interactive effects are of less interest because public health interventions aimed at reducing one exposure would likely reduce the other exposures.

*What is the health effect of cumulative chemical exposure?* A third question estimates the association between cumulative chemical exposure and human health. Here we are trying to quantify the summary effect of a class or multiple classes of exposure. Unlike the question of interaction, we assume that joint exposure to the chemicals does not have a greater than additive effect on the outcome (in the toxicological sense) and that we can meaningfully condense the different exposures into a single summary metric. This may be most appropriate and insightful when the individual components of the mixture act via common biological pathways (e.g., phthalates or dioxins), when the exposure to individual agents is below some threshold of concern [e.g., no-observed-adverse-effect level (NOAEL) or lowest-observed-adverse-effect level (LOAEL)], and when there are individuals whose aggregate exposure is over this threshold.

Summaries of cumulative exposure can include simple summations of the concentration of individual exposures or by weighting them according to their biological potency [e.g., toxic equivalency factors (TEFs) for dioxin-like compounds] (Burns et al. 2011; Safe 1998). Although simple summary measures such as total serum PCB concentrations can be used, they often reflect the individual component with the highest concentration in the mixture (Axelrad et al. 2009). Thus, these summary measures may not accurately capture the cumulative effect of the mixture if the lower concentration components are more potent than the higher concentration ones. As an alternative, more complex weighting approaches can be used when making certain assumptions about the underlying biology of the dose–response relationship (e.g., concentration addition). One limitation to this approach is that epidemiologists will often require toxicological data that quantifies the biological activity of individual components of the mixture (e.g., TEFs for dioxin-like compounds). Furthermore, different health end points (e.g., cancer vs. neurodevelopment) may need different summary measures or weights to accurately describe the cumulative exposure to the mixture.

There are several additional strategies that can be used to estimate the cumulative health effects of a mixture. One could quantify the total biological activity in individual biospecimens through integrative assays (e.g., total estrogenicity) and use it as a measure of exposure (Howard and Webster 2013; Vilahur et al. 2013). These measures have the advantage of capturing both additive and interactive effects. Statistically driven approaches, such as principal components analysis, can identify latent factors that explain the correlation between mixture components. These factors can be used as an exposure variable in statistical models (Maresca et al. 2015). Although principal components methods are advantageous for studying some exposures, particularly those with common sources (e.g., air pollution), the derived factors are difficult to interpret because they are on a dimensionless scale that is not specific to any one chemical exposure, and they may be unique to the population being studied, thus limiting their generalizability. Other methods, including empirically estimated weights, may be used to create weighted sums of standardized concentrations (Czarnota et al. 2015).

## Conclusions

By defining the types of research questions related to chemical mixtures that epidemiological studies can address, we hope to identify the gaps in our knowledge and develop or apply appropriate statistical methods that accurately quantify the impact of chemical mixtures on human health. In this article, we have chosen to focus on environmental chemicals, but the three questions we describe naturally extend to other environmental exposures (e.g., air pollution and infectious agents), as well as the broader exposome (e.g., stress and nutrition) (Wild 2005). By examining chemical mixtures, instead of one chemical at a time, we may identify risk factors for diseases with environmental origins and develop more targeted public health interventions.
